# Public Recognition of Emergencies and Appropriate Ambulance Use in Riyadh: A Cross-Sectional Survey

**DOI:** 10.3390/healthcare13212801

**Published:** 2025-11-04

**Authors:** Meshary S. Binhotan, Ghadah Alhammad, Abdullah N. Alshibani, Abdulrhman S. Alghamdi, Abdullah A. Alabdali, Ahmed M. Alotaibi, Meshal E. Alharbi

**Affiliations:** 1 Emergency Medical Services Department, College of Applied Medical Sciences, King Saud bin Abdulaziz University for Health Sciences, Riyadh 11481, Saudi Arabiaabdalia@ksau-hs.edu.sa (A.A.A.);; 2 King Abdullah International Medical Research Center, Riyadh 11481, Saudi Arabia; 3 Emergency Medical Services and Dispatch Department, Saudi Response Plus Medical, Riyadh 14251, Saudi Arabia; g.i.alhammad@gmail.com

**Keywords:** public awareness, public safety, emergency cases recognition, EMS utilization, public health

## Abstract

**Background/Objectives**: The demand for Emergency Medical Services (EMS) has increased over the past few years, increasing the EMS burden. Public utilization of EMS for non-emergency cases is a major global issue contributing to this burden. This study explores the public’s ability to accurately recognize emergency situations and appropriately request ambulance services. **Methods**: This cross-sectional study utilized a survey to explore public awareness among residents of Riyadh, Saudi Arabia. The survey was developed from the relevant literature and panel discussions, followed by validation through a pilot study. Recruitment was conducted in different publicly accessible places to capture the diverse demographics of the residents. The sample size was statistically calculated using the Raosoft sample size calculator to identify significant differences. **Results**: This study included 522 respondents, predominantly females (79%) aged 18–34 (42%) and 35–54 (41%) years. Both males and females correctly identified around two-thirds of the total emergency cases, with means of 6.49 (SD = 1.27) and 6.55 (SD = 1.32), respectively. Appropriate ambulance requests were made in less than one-third of the emergencies by both males and females, with means of 2.29 (SD = 1.29) and 2.38 (SD = 1.32), respectively. Stroke and older adults with hip pain were the most accurately recognized emergency cases at 92.5% and 90%, respectively, while mild chest pain and child head hematoma were the least accurately recognized at 36.6% and 38.5%. Women in labor and objects in the ear canal were the most misidentified as emergencies at 97.3% and 87.7%, respectively. **Conclusions**: This study highlights the prevalence of unrecognized emergency situations and the underutilization of EMS for real emergency cases. The findings recommend the need for national training programs and provide valuable insights for EMS dispatcher training programs regarding public perceptions of emergency and non-emergency situations. While the findings provide insights into targeted preventive measures to alleviate the EMS burden, they also demonstrate the critical role of public awareness in enhancing public health safety concerning emergency situations.

## 1. Introduction

The demand for Emergency Medical Services (EMS) has been increasing over the past few years, placing significant stress on these services [1,2]. This heightened demand necessitates further human and financial resources to alleviate the burden. In Saudi Arabia, requests for Saudi Red Crescent Authority (SRCA) services—the main EMS provider across the country—have significantly increased, exceeding the population growth rate [1,3]. This demand increased by 28.96% from 2017 to 2019 and by 47.03% from 2019 to 2020 [1,3]. Such high demand can influence patients’ access to healthcare services, as it exceeds the available supply. Furthermore, this situation can exacerbate the burden on EMS and hospital emergency departments which, in turn, increases response times and decreases quality of care, negatively impacting patient outcomes [1,4,5].

Several factors have been identified as contributing to raising EMS demand. Changes in population demographics, with a notable increase in the elderly population, accompanied by different co-morbidities have influenced the surge in requests for EMS [3]. A lack of social support, including inadequate transportation for patients to hospitals, and social isolation have been found to increase the EMS workload [6,7]. In addition, delays faced by EMS providers during patient handovers at emergency departments, due to the overcrowding of these departments, have increased the time spent on each EMS call, subsequently extending the utilization period of EMS [1,6,7]. Other contributing factors include ease of access to ambulance services and increased community awareness of health services [6]. The utilization of EMS for non-emergency situations represents a significant and avoidable factor that puts additional strain on these services.

The public utilization of EMS for non-emergency situations represents a significant global issue, placing an unnecessary burden on these services [8,9]. However, the extent of this burden varies by country. For instance, in Sweden, cases that did not need actual ambulance services accounted for one-third of the total EMS requests [8], whereas in some regions of England, it was reported that 16% of EMS-transported cases to emergency departments were not urgent [9]. Although there is a lack of studies regarding non-emergency EMS calls in Saudi Arabia, a hospital-based EMS in Jubail found that 47% of the calls were for non-urgent matters such as regular appointments and patients on dialysis [10]. Moreover, the effects of such cases are not merely limited to occupying medical services and facility; they also have financial implications, potentially costing more than real emergency calls [10]. The unnecessary burden on EMS, driven by non-urgent cases and coupled with the increasing demand for EMS [1], necessitates immediate intervention to achieve a balance between EMS demand and supply.

Raising public awareness of when to seek EMS can significantly influence the appropriate utilization of the service. Two studies conducted in Australia found that the general public often struggle to identify situations that appropriately require EMS intervention [11,12]. Cases such as early labor stages, Lego in the ear, and flu—none of which are true emergencies—can be considered as unnecessary EMS requests [12]. Thus, assessing public understanding of emergency versus non-emergency situations can shed light on factors contributing to the increased EMS demand. This knowledge can inform the development of preventive strategies, including public awareness programs. However, the existing literature reveals a scarcity of studies exploring the public’s ability to differentiate between emergency and non-emergency cases and to appropriately call for ambulance services [11,12,13]. In Saudi Arabia, this lack of knowledge has not yet been examined, highlighting a significant knowledge gap.

This study aims to investigate public knowledge on the recognition of emergency situations and the decision-making process involved in requesting an ambulance service in Saudi Arabia. A validated questionnaire was distributed among members of the public residing in Saudi Arabia to assess their ability to correctly identify emergency and non-emergency cases, and their understanding of when to utilize EMS. The findings are expected to highlight deficiencies in public knowledge regarding the correct identification of emergencies and appropriate EMS utilization, providing insights for researchers and policymakers in guiding future research and informing preventative strategies. Addressing these gaps could enhance the efficiency of EMS utilization and reduce the work burden on EMS personnel and the cost of EMS overutilization. We hypothesize that most of the public can correctly recognize emergency scenarios and appropriately call for ambulance services. We also hypothesize that there are differences in responses to emergency and non-emergency scenarios based on participants’ demographics.

## 2. Materials and Methods

### 2.1. Study Design and Settings

This is a cross-sectional study that employed a survey to assess public awareness regarding emergency and non-emergency cases. It was conducted in Riyadh, the capital of Saudi Arabia, where the population is 7,009,129, of which 3,657,768 are non-Saudi nationals [14]. This study recruited participants from November to December 2023, during which the calculated sample size was achieved. This study included adults (≥18) who speak and read Arabic or English, in order to maximize the chances of capturing the knowledge of both Saudi and non-Saudi nationals.

### 2.2. Sample Size Calculation

The sample size was statistically calculated using the Raosoft^®^ (Raosoft, Inc., Seattle, WA, USA) sample size calculator to identify significant differences. Based on the latest Saudi census data, a margin error of 5%, and a confidence level of 95%, the required sample size was 385.

### 2.3. Materials

This study utilized a survey developed based on existing research on similar topics and expert panel discussions. A previously validated survey in different settings was initially used to develop the current survey [11,12,13]. This survey comprised 17 medical and trauma scenarios—9 for emergency and 8 for non-emergency—that were randomly arranged. To optimally assess the public awareness in Saudi Arabia, the survey was revised to reflect the Saudi culture and prevalent case types. A panel of expert EMS providers, including three EMS assistant professors and one paramedic with over 10 years of clinical and educational experience, reviewed the scenarios, resulting in the replacement of two scenarios and the modification of three responses. The inter-rater agreement (Supplementary File S1) was calculated using Fleiss’s kappa; the overall κ = 0.78, indicating substantial agreement among the experts. These modifications were based on either cultural considerations (replacing the ‘alcohol intoxication’ scenario with a ‘no appetite’ scenario) or the low likelihood of occurrence (replacing the ‘box jellyfish sting’ scenario with a ‘seizure’ scenario). In addition, the responses were modified to reflect the healthcare setting in Saudi Arabia (such as replacing ‘call 000 for an ambulance’ with ‘call 997 for an ambulance’). The revised survey was translated into Arabic, the spoken language in Saudi Arabia, by two bilingual EMS providers fluent in both languages to ensure a comprehensive understanding of the survey. The Arabic version was then back-translated into English to ensure comprehensive understanding and equivalence of meaning.

After developing the revised survey, a pilot study was conducted to ensure the validity of the new version. The final draft of the survey was distributed to another three expert EMS providers with more than 10 years of experience and a sample of the public. They were instructed to review the clarity, including understandability and readability, of the survey and to offer suggestions for improvement. Based on their feedback, minor modifications were made to enhance the readability of the final draft, leading to the creation of the final version of the survey. This final version was agreed upon by the panel members through consensus and subsequently utilized in this study (Supplementary File S2).

### 2.4. Survey Scenarios

The survey comprised 17 hypothetical scenarios, of which 9 were emergencies and 8 were not (Table 1 and Table 2). Each scenario was presented with nine responses, where participants were allowed to choose only one response for each scenario. The nine responses included the following: (1) call 997 for an ambulance (997 is the Saudi ambulance service hotline), (2) go to the emergency department, (3) make an appointment to visit a general physician/primary healthcare provider, (4) talk to a pharmacist, (5) make an appointment at a COVID-19 clinic, (6) call the medical consultation service (937), (7) provide first aid, (8) no immediate action but monitor the situation, and (9) other.

Two of the responses—namely, ‘call 997 for an ambulance’ and ‘go to the emergency department’—indicate that the case is recognized as an emergency. The other responses suggest a non-emergency identification. Only ‘call 997 for an ambulance’ was used to assess the participant’s ability to correctly request an ambulance for any of the nine emergency scenarios.

### 2.5. Demographics

In the survey, participants were initially required to complete a demographics section. The demographics section encompassed questions on gender, age, nationality, financial income, educational level, whether they have worked or studied in the medical field, if they have a relative working in the medical field, and whether they have watched medical TV series. These variables were used to identify potential contributing factors for correct emergency recognition and appropriate EMS utilization.

### 2.6. Procedure

The digital version of the survey and printed copies were distributed in various publicly accessible places throughout the city of Riyadh. The places were previously chosen to capture different demographics of Riyadh residents. The places included large shopping malls, schools, metro stations, and universities. The potential participants were approached during early day time (09:00–12:00) and early evening (17:00–21:00), during both weekdays and weekends. All participants were informed of this study’s aim and were assured that their participation was voluntary, with no incentives offered. The participants were allowed to choose their preferred language for the survey, either Arabic or English. Prior to taking part in this study, all participants signed the consent form and were instructed to imagine the scenario as a real-life situation and to answer the questions accordingly.

### 2.7. Statistical Analysis

The data were first checked for normality using the Shapiro–Wilk test. Descriptive analysis using mean ± standard deviation was conducted to present continuous variables, and frequencies were used for categorical variables. These findings addressed the main objective concerning assessing public awareness. To compare responses to emergency and non-emergency scenarios across participants’ demographics, an independent t-test was used for gender, nationality, and variables including studying or working in the medical field, having a relative working in the medical field, and watching medical TV series, while an analysis of variance (ANOVA) test was used for age, financial income, and level of education. Moreover, 95% confidence intervals (CIs) were reported for recognizing emergencies and calling for an ambulance, and were measured as primary estimands. To identify predictors of correctly recognizing emergency cases and appropriately calling for an ambulance, multiple linear regression was employed. The covariates added were gender, nationality, age in years, income in SAR, education level, studying or working in the medical field, having a relative working in the medical field, and watching medical TV series. Multicollinearity was tested using Variance Inflation Factors (VIF) and tolerance factors. All data were analyzed using IBM SPSS Statistics (IBM Corp., Armonk, NY, USA), version 30.

### 2.8. Ethical Considerations

Ethical approval from the Institutional Review Board (IRB) at King Abdullah International Medical Research Centre was received (NRC23R/625/10 October 2023) to conduct this study. Informed consent was electronically attached to the survey to be signed by participants prior to starting the questionnaire. No personal identification data were collected, and all participants were assigned an anonymous number, to ensure the privacy of the participants. All data were stored on a password-protected computer at the Emergency Medical Services Department in King Saud bin Abdulaziz University for Health Sciences, supervised by the primary investigator and accessed only by the research team. These data are not openly available but are available on request from the corresponding author. All collected data follow the retention policy by King Abdullah International Medical Research Center, allowing three years of data storage. All ethical procedures were strictly followed throughout the study procedure to guarantee the anonymity and confidentiality of the collated data.

## 3. Results

This study analyzed 522 responses from individuals residing in Riyadh. Most participants were females (79%), and Saudi was the predominant nationality (92%) (see Table 3). The majority of the participants were in the age groups of 18–34 (42%) and 35–54 (41%). Most participants received a constant income, which peaked at 22.6% for those earning SAR < 5000 (USD < 1333) and 19.7% for those earning between SAR 5000 and 10,000 (USD 1333–2666), while 16.3% had no stable income. In terms of education, 68.4% of the participants held a bachelor’s degree, whereas only 20% had a high school certificate. While 86% of respondents did not work or study in the medical field, a significant 69% had family members employed in healthcare, and nearly half (49.4%) regularly watched medical TV series.

Table 3 presents a comparison of the participants’ responses according to their demographics. Both males and females accurately identified around two-thirds of the total emergency cases, with means of 6.49 (SD = 1.27) and 6.55 (SD = 1.32), respectively, with no significant difference. However, males significantly demonstrated a greater ability to recognize non-emergency scenarios compared to females (*p* = 0.006). Saudi and non-Saudi respondents performed similarly in recognizing emergency cases, with means of 6.52 (SD = 1.30) and 6.63 (SD = 1.37), respectively; however, no significant difference was noted. Conversely, a significant difference (*p* = 0.001) was observed in non-emergency cases where non-Saudi participants exhibited better ability compared to Saudi participants, scoring means of 5.07 (SD = 1.35) and 4.24 (SD = 1.24), respectively. The accuracy of recognizing emergency increased with age, peaking at an average score of 6.64 (SD = 1.29) for individuals over 55 years, albeit with no significant difference. In contrast, the recognition of non-emergency cases significantly declined with age, with participants aged 18–34 being the most proficient, achieving a mean score of 4.59 (SD = 1.25, *p* = 0.001). Financial income showed no significant difference in recognizing emergency cases, with means ranging from 6.42 to 6.61. For non-emergency cases, participants earning more than SAR 20,000 (USD 5333) had significantly the lowest mean score at 3.83 (SD = 1.30), whereas those earning less than SAR 5000 (USD < 1333) scored highest, with a mean of 4.75 (SD = 1.18, *p* = 001). No significant differences were noted across other demographics factors. The Bonferroni correction was performed to control for multiple comparisons for both emergency and non-emergency scenarios, resulting in an adjusted significance *p* value <0.003. After the Bonferroni correction, significant differences were detected for age (*p* = 0.001), nationality (*p* = 0.001), and income (*p* = 0.001) for non-emergency scenarios. There were no statistical differences in the emergency scenario following the Bonferroni adjustment.

Table 4 presents the frequency of emergency cases correctly and incorrectly identified by the participants. Stroke and older adults with hip pain were the highest emergency cases correctly recognized as emergencies by the respondents, at 92.5% and 90%, respectively. Conversely, mild chest pain and child head hematoma were the lowest, with only 36.6% and 38.5%, respectively. On the other hand, woman in labor and Lego in ear canal were the two highest cases falsely recognized as emergencies, at 97.3% and 87.7%, respectively.

**Figure 1:**
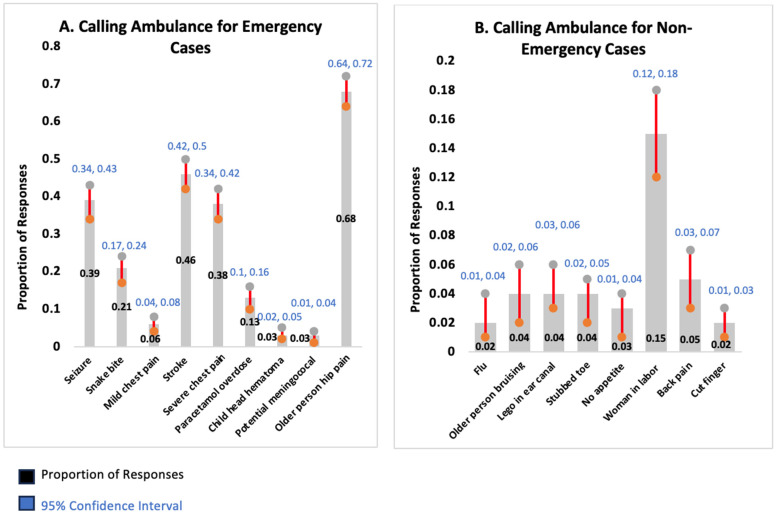
Proportion of responses with 95% confidence interval (CI) for ambulance services utilization across emergency (**A**) and non-emergency (**B**) cases.

Further analysis assessed the ability of participants to recognize the need to call for an ambulance in emergency cases. Participants called for an ambulance in less than one-third of these cases, with no statistically significant differences related to participants’ demographic factors (see Table 5). Among the emergency cases, older adults with hip pain was the most appropriately requested situation for an ambulance, followed by stroke, at 68% and 46.2%, respectively (see Table 6 and Figure 1). Conversely, potential meningococcal disease and child head hematoma were the two lowest, at 2.7% and 3.4%, respectively. For non-emergency cases, women in labor represented the most common situation incorrectly classified as requiring an ambulance, at 14.9%.

## 4. Discussion

This study aimed to assess the public’s ability to identify emergency situations and their subsequent decision-making in calling for ambulance services. It also explored factors that may influence their ability to recognize emergency cases and appropriately utilize ambulance services, in order to identify potential areas for improvement. The key findings showed that around two-thirds of participants correctly identified the emergency cases, with no significant differences based on sociodemographic factors. However, only about half of the participants accurately recognized non-emergency scenarios as low-acuity cases. Male, non-Saudi nationality, younger age, and earning a salary of less than SAR 5000 (USD < 1333) were significantly associated with improved recognition of non-emergency cases. Recognition rate varied across the different emergency cases, with stroke and older adult with hip being identified frequently, while mild chest pain and child head hematoma were the least. Similarly, misinterpreting non-emergency cases as emergencies was varied, with woman in labor and Lego in ear canal being the most commonly misidentified cases. Less than one-third of participants appropriately called EMS for true emergency cases, with the highest utilization for the case of older adults with hip pain. Conversely, the highest number of inappropriate EMS calls were for woman in labor as the most common non-emergency case used to request an ambulance, emphasizing a need to enhance public awareness of such cases. These findings provide valuable insights for public health sectors and health education institutions, suggesting potential areas for improvement to avoid the unnecessary burden on human and financial resources in EMS.

The majority of participants—around two-thirds—correctly recognized emergency situations, demonstrating an above-average performance. However, nearly one-third underestimated the emergency cases as non-emergencies. These findings fall short of the findings found in different contexts in Australia [11], requiring further enhancement of public knowledge. No significant differences in recognition were observed across the sociodemographic factors regarding emergency situations, including gender and age, indicating an equivalent level of awareness. Other studies have reported similar findings; for example, females were linked to higher accuracy in identifying emergency cases, although this was not statistically significant [11]. Conversely, other research has shown significance between males and appropriate emergency department visits, potentially reflecting better identification of emergency cases [15]. Although age was not shown to be a factor influencing recognition of emergency cases [11], other findings revealed age (60 years and over) as a strong predictor of appropriate emergency department visits, likely due to better identification of emergency cases stemming from life experiences [15]. In addition, having a healthcare background or first aid training has been shown to be significantly associated with correct identification of emergency cases [11], likely attributed to the previous medical knowledge these individuals possess. Though our findings mirrored these trends—females, older adults, and participants with a medical background scored higher in identifying emergency cases—they were not statistically significant in our study. These observed variations may be attributed to different factors, including disparities in educational systems and cultural contexts across different regions globally. Thus, it is recommended that each public health sector undertake a surveillance study to evaluate the level of emergency awareness among the public and to identify both national and local gaps for improvement.

For non-emergency cases, our findings revealed that around half of the participants correctly recognized the non-emergency cases as low-acuity cases, indicating that half of the non-emergency cases were mistakenly perceived as emergencies. This substantial misinterpretation of non-emergency cases could lead to unnecessary urgent actions, such as calling EMS or visiting emergency departments. Such attitudes could increase the strain on EMS and emergency departments, thereby reducing time-sensitive responses to real emergencies, including life-threatening cases [4,5].

Our findings showed that certain demographics—male, non-Saudi national, younger adult, and monthly earnings of less than SAR 5000 (USD < 1333)—were significantly associated with better performance in accurately identifying such cases. With regard to gender, the existing literature presents mixed conclusions. Some studies were consistent with our findings, showing that females are more likely than males to call for an ambulance [16] and to attend emergency departments [17,18] for non-emergency cases, reflecting an overestimation of non-urgent cases. However, others reported no significant differences between genders [13,19,20], with some indicating that females are less likely to seek emergency care for non-emergency conditions [12]. In terms of age, our findings align with previous studies demonstrating that older adults are more likely than younger adults to misinterpret non-emergency cases and seek emergency care [12,21]. Nevertheless, some research has reported that younger adults are more likely to pursue emergency care for non-emergency cases [17,22] or there are no significant differences between age groups [13,19]. A possible explanation for the inappropriate utilization of emergency services could be the convenience and accessibility of these services compared to non-emergency care services [17,23]. High household income was shown in a recent study to be associated with better identification of non-emergency cases [12], which is contrary to our findings that indicated a significant association between low monthly income (less than SAR 5000) and improved identification of these cases. These differences in the wider literature can reflect the variations in, for example, healthcare systems where EMS or emergency departments are easily accessible, some educational systems which implement Basic Life Support (BLS) courses within their school curriculum, and other public health initiatives [6,24,25,26,27].

Our analyses revealed variations in the frequency of identifying different sets of emergency and non-emergency scenarios. Stroke and older adults with hip pain were the most accurately identified emergency cases, consistent with the broader literature that reports a high recognition rate for these cases [11,13]. Severe chest pain was identified by almost all participants as a case of emergency [11], but it had a low identification rate in our study (36.6%). This low identification could be attributed to the misperception of severe chest pain as a non-emergency, stemming from a low awareness of these symptoms and their critical nature [28]. A previous study conducted among the Middle Eastern population indicated a significant lack of awareness of heart attack symptoms, where people often attribute chest pain and discomfort to fatigue or indigestion rather than recognizing them as signs of an emergency cardiac condition [29]. This substantial underestimation of severe chest pain as an emergency necessitates immediate intervention, including targeted training programs, as early treatment of heart attack patients is significant for their survival and recovery [30,31].

Among non-emergency cases, the cut finger was the most accurately identified non-emergency situation, mirroring similar findings elsewhere [11,13]. However, the woman in labor and Lego in ear canal cases were the most commonly misrecognized as emergencies in our study, though both are often perceived as emergencies in the literature [11,13]. This misinterpretation could be influenced by the associated anxiety of these situations. For example, the breaking of water in pregnant women is perceived by many as a sign of urgency, regardless of whether labor has started or not [11]. Various studies have also highlighted the differences in knowledge regarding people’s perceptions of the actual signs of pregnancy complications due to several factors including cultural and sociodemographic factors [32,33,34]. Enhancing these perceptions could improve the recognition of genuine emergencies and influence subsequent actions, including unnecessary emergency department visits and EMS utilization. These noted differences in the recognition of emergencies and non-emergencies across communities underscore the need for targeted public education programs aimed at improving awareness across varying situations, particularly those most prevalent within each community.

EMS were appropriately utilized in less than one-third of the emergency cases, highlighting significant shortcomings in decision-making during emergencies, considering that two-thirds of emergency cases were correctly recognized. This underutilization may stem from a lack of awareness of EMS roles and perceived belief that ambulances will take a long time to arrive [35,36,37]. The tendency to go directly to emergency departments rather than calling an ambulance could also be motivated by the belief that one would receive faster treatment for critical conditions [38]. Consistent with the pattern of highly recognized emergency cases, older adults with hip pain and stroke were the most common cases for which participants requested an ambulance. These findings are similar to those from other studies highlighting cases in which EMS were highly requested [11]. However, severe chest pain emerged as the most commonly reported emergency case requiring the ambulance service (92.8%), in contrast to our findings (37.7%) [11]. The lowest rate of appropriate EMS utilization in our study was for child head hematoma (3.4%) and potential meningococcal disease (2.7%), reflecting a similar pattern to a previous study that reported 1% for child head hematoma and 6% for potential meningococcal disease [11]. These low rates could be attributed to the lack of awareness of the seriousness of these conditions [11,29]. For non-emergency cases, respondents indicated a higher likelihood of calling an ambulance for a woman in labor, mirroring similar findings from the wider literature [11,12,13]. Conversely, flu and cut fingers were the least frequent non-emergency cases for which an ambulance was requested, consistent with broader investigations [11,12,13]. However, some studies suggested a higher proportion of calling an ambulance for flu cases [12], likely due to the fear of flu during the COVID-19 pandemic [39]. These findings could offer insights for EMS systems, suggesting that dispatchers should thoroughly investigate cases which the public is likely to misinterpret as emergencies and, subsequently, prioritize the response to real emergencies.

Our findings provide several insights for policymakers and recommend directions for future research. The overall lack of public awareness in identifying emergency cases underscores the need for national initiatives to enhance the public’s ability to identify such cases. The identified demographic factors—notably, age and gender—influencing the ability to differentiate between emergency and non-emergency cases indicate a demand for tailored educational programs to increase their effectiveness. Some of our findings diverge from those observed in other contexts, such as Australia, emphasizing the significance of each country conducting a national surveillance study to assess the awareness levels among their populations and to subsequently develop preventive strategies. The identified non-emergency cases for which participants often called ambulances can inform EMS dispatcher training programs to enhance call negotiation and improve the ability to distinguish between real emergencies and non-emergencies. While a significant number of participants correctly identified emergency cases, many of them preferred to go to emergency departments rather than calling an ambulance. Therefore, a further study is recommended to investigate public preferences for different modes of transportation (calling for an ambulance vs. self-transport to an emergency department) in both emergency and non-emergency situations, in order to reveal the underlying reasons and formulate recommendations for optimizing the use of EMS. Finally, the misperception of non-emergency cases as emergencies and the calling of ambulances for non-emergencies found in our study require further research, with qualitative findings to investigate public perceptions of these cases and to inform the development of comprehensive training programs.

This study has several strengths that should be highlighted. It evaluated the ability of public residents to recognize emergency cases and subsequently request ambulance services, providing a comprehensive data set that highlights deficiencies in the emergency response cycle. The survey used in this study was validated through a pilot study to ensure the accurate measurement of the study objectives. Nonetheless, certain limitations should also be acknowledged. First, this study had a higher representation of females and Saudi respondents compared to the actual demographics of Riyadh residents (Table A2). However, the recruitment of participants was conducted in publicly accessible places, and individuals from all demographics were invited to voluntarily take part in this study. Second, this study was conducted in Riyadh, which may restrict the generalizability of the findings to other contexts. Nevertheless, this is the first study conducted within a Saudi context, providing valuable insights to address the gap in understanding the recognition of emergency situations and the appropriate utilization of EMS.

## 5. Conclusions

This study assessed the public’s ability to recognize emergency cases and appropriately request EMS. Around two-thirds of the emergencies were correctly recognized, while only about half of non-emergencies were accurately identified, underscoring the need for public awareness campaigns. Demographic factors such as gender and age revealed significant differences in identifying non-emergency situations, suggesting the necessity for tailored educational programs. Moreover, participants demonstrated different levels of recognition for specific emergency and non-emergency situations, highlighting the need for more comprehensive training programs, with a particular focus on the most prevalent real-life emergency cases. EMS were appropriately utilized in less than one-third of the emergency cases, highlighting the importance of an enhanced public awareness of these services. These findings provide valuable insights into strategies for enhancing public awareness and appropriate EMS utilization which, in turn, have the potential to save lives and reduce the burden on EMS.

## Figures and Tables

**Table 1:** List of emergency scenarios provided to the participants.

Scenario No.	Scenario Title	Scenario Description
1	Seizure	Your 14-year-old brother is experiencing a seizure for the first time in his life.
3	Snake bite (unidentified)	A woman aged 50 years has been bitten by an unidentifiable snake on her ankle.
5	Mild chest pain	A woman aged 40 years is complaining of mild chest pain that she does not think is indigestion or related to muscle strain.
8	Stroke	A man aged 67 years awoke complaining of limb numbness, walking difficulties, and slurred speech; he has not consumed any alcohol.
9	Severe chest pain	A man aged 52 years is experiencing severe chest pain, and he is sweating and gray in color.
13	Paracetamol overdose	A female aged 32 years has taken 10 tablets of paracetamol regularly over the last 12 h, and she is feeling extremely unwell. She has abdominal pain and nausea.
15	Child head hematoma	A 3-year-old boy fell from a couch and bumped his head. He immediately started crying and developed a golf-ball-sized lump with a bruise.
16	Potential meningococcal disease	A 4-year-old girl awoke with fever, feels hot to the touch, and has a sore neck and headache that have not been relieved with Panadol.
17	Older person hip pain	A woman aged 80 years fell out of her bed and is now unable to stand up, experiencing right-side hip pain.

**Table 2:** List of non-emergency scenarios provided to the participants.

Scenario No.	Scenario Title	Scenario Description
2	Flu	A man aged 45 years is experiencing flu-like symptoms. He has a mild fever, headache, runny nose, and fatigue.
4	Older person bruising	A woman aged 77 years had an immediate large bruise on her thigh after bumping into the kitchen table.
6	Lego in ear canal	A 4-year-old girl has a piece of Lego stuck in her ear canal.
7	Stubbed toe	A man aged 25 years plays football with his bare feet in a backyard with his friends, and he stubs his toe on a brick. There is blood and it is throbbing painfully.
10	No appetite	A teenager with no past medical history has nausea this afternoon, has not eaten since yesterday, and refuses to eat due to a lack of appetite.
11	Woman in labor	A 33-year-old woman who is 9 months pregnant goes into the early stages of labor. Her uterus’ water has broken, and she is uncomfortable.
12	Back pain	A 40-year-old man with back pain for the last 6 months wakes up at night with a sore back and has no painkillers left. He is in pain.
14	Cut finger	A man aged 42 years cuts his finger while chopping vegetables. He has bleeding which is controlled with applied pressure.

**Table 3:** Comparing responses to emergency and non-emergency scenarios across participants’ demographics.

Details	Number (%)	Emergency Scenario			Non-Emergency Scenario		
		Mean 0–9 (SD)	95% CI	*p* Value	Mean 0–8 (SD)	95% CI	*p* Value
**Gender ^a^**							
Males	109 (20.9)	6.49 (1.27)	(6.25, 6.73)	0.665	4.61 (1.28)	(4.36, 4.85)	0.006 *
Females	413 (79.1)	6.55 (1.32)	(6.42, 6.67)		4.23 (1.26)	(4.11, 4.36)	
**Nationality ^a^**							
Saudi	478 (91.6)	6.52 (1.30)	(6.41, 6.64)	0.589	4.24 (1.24)	(4.13, 4.35)	0.001 *
Non-Saudi	44 (8.4)	6.63 (1.37)	(6.22, 7.05)		5.07 (1.35)	(4.66, 5.48)	
**Age ^b^**							
18–34	221 (42.3)	6.42 (1.27)	(6.25, 6.59)	0.23	4.59 (1.25)	(4.42, 4.75)	0.001 *
35–54	214 (41)	6.61 (1.35)	(6.43,6.79)		4.12 (1.32)	(3.94, 4.29)	
55+	87 (16.7)	6.64 (1.29)	(6.37, 6.92)		4.08 (1.07)	(3.85, 4.31)	
**Financial Income ^b^ (SAR)**							
Without a constant monthly income	85 (16.3)	6.61 (1.40)	(6.31, 6.91)	0.914	4.40 (1.43)	(4.09, 4.71)	0.001 *
Less than 5000	118 (22.6)	6.42 (1.32)	(6.18, 6.66)		4.75 (1.18)	(4.53, 4.96)	
5000–10,000	103 (19.7)	6.60 (1.31)	(6.35, 6.86)		4.31 (1.16)	(4.08, 4.54)	
10,001–15,000	86 (16.5)	6.56 (1.24)	(6.29, 6.82)		4.20 (1.18)	(3.94, 4.45)	
15,001–20,000	72 (13.8)	6.50 (1.30)	(6.19, 6.81)		4.01 (1.25)	(3.72, 4.31)	
More than 20,000	58 (11.1)	6.53 (1.27)	(6.20, 6.87)		3.83 (1.30)	(3.49, 4.17)	
**Education ^b^**							
High School	108 (20.7)	6.73 (1.26)	(6.49, 6.97)	0.206	4.27 (1.27)	(4.03, 4.51)	0.899
Bachelor’s	357 (68.4)	6.47 (1.32)	(6.34, 6.61)		4.33 (1.25)	(4.20, 4.46)	
Higher Studies	57 (10.9)	6.53 (1.30)	(6.18, 6.87)		4.28 (1.40)	(3.91, 4.65)	
**Do you study or work in the medical field? ^a^**							
Yes	72 (13.8)	6.61 (1.18)	(6.33, 6.89)	0.593	4.44 (1.38)	(4.12, 4.77)	0.335
No	450 (86.2)	6.52 (1.33)	(6.40, 6.64)		4.29 (1.25)	(4.12, 4.77)	
**Do you have a relative working in the medical field? ^a^**							
Yes	362 (69.3)	6.55 (1.34)	(6.41, 6.69)	0.636	4.30 (1.27)	(4.18, 4.44)	0.979
No	160 (30.7)	6.49 (1.22)	(6.30, 6.68)		4.31 (1.28)	(4.11, 4.51)	
**Do you watch medical TV series? ^a^**							
Yes	258 (49.4)	6.55 (1.28)	(6.39, 6.70)	0.836	4.33 (1.30)	(4.17,4.49)	0.683
No	264 (50.6)	6.52 (1.33)	6.36, 6.68)		4.29 (1.24)	(4.14, 4.44)	

* Statistically significant at 5%; **^a^** independent *t*-test; **^b^** ANOVA test.

**Table 4:** Frequency of cases correctly and incorrectly recognized as emergencies by the participants (*N* = 522).

Emergency Case	Correctly Recognized as Emergency (%)	Non-Emergency Case	Incorrectly Recognized as Emergency (%)
Seizure	347 (66.5)	Flu	161 (30.8)
Snake bite	451 (86.4)	Older person Bruising	122 (23.4)
Mild chest pain	191 (36.6)	Lego in ear canal	458 (87.7)
Stroke	483 (92.5)	Stubbed toe	287 (55)
Severe chest pain	453 (86.8)	No appetite	132 (25.3)
Paracetamol overdose	431 (82.6)	Woman in labor	508 (97.3)
Child head hematoma	201 (38.5)	Back pain	160 (30.7)
Potential meningococcal disease	384 (73.6)	Cut finger	98 (18.8)
Older person hip pain	470 (90)	-	-

**Table 5:** Demographic characteristics of participants who correctly called for an ambulance.

Variables	Mean 0–9 (SD)	95% CI	*p* Value
**Gender ^a^**			
Males	2.29 (1.29)	(2.04, 2.53)	0.52
Females	2.38 (1.32)	(2.26, 2.51)	
**Nationality ^a^**			
Saudi	2.36 (1.31)	(2.24, 2.48)	
Non-Saudi	2.41 (1.39)	(1.99, 2.83)	0.821
**Age (in years) ^b^**			
18–34	2.45 (1.36)	(2.27, 2.63)	
35–54	2.32 (1.27)	(2.15, 2.49)	0.416
55 and above	2.26 (1.33)	(1.98, 2.55)	
**Income ^b^**			
Without a constant monthly income	2.58 (1.47)	(2.26, 2.89)	
Less than 5000	2.28 (1.23)	(2.05, 2.50)	
5000–10,000	2.49 (1.36)	(2.23, 2.76)	0.323
10,001–15,000	2.20 (1.21)	(1.94, 2.46)	
15,001–20,000	2.24 (1.40)	(1.91, 2.56)	
More than 20,000	2.41 (1.21)	(2.09, 2.73)	
**Education ^b^**			
High School	2.49 (1.20)	(2.26, 2.72)	0.47
Bachelor’s	2.32 (1.34)	(2.18, 2.46)	
Higher Studies	2.42 (1.41)	(2.05, 2.80)	
**Do you study or work in the medical field? ^a^**			
Yes	2.51 (1.29)	(2.21, 2.82)	
No	2.34 (1.32)	(2.22, 2.46)	0.305
**Do you have a relative working in the medical field? ^a^**			
Yes	2.43 (1.35)	(2.29, 2.57)	
No	2.22 (1.22)	(2.03, 2.41)	0.09
**Do you watch medical TV series? ^a^**			
Yes	2.44 (1.32)	(2.28, 2.60)	0.217
No	2.29 (1.31)	(2.14, 2.45)	

**^a^** Independent *t*-test; **^b^** ANOVA test; significance level at 5%.

**Table 6:** Participants’ responses (*N* = 522) to calling an ambulance among emergency and non-emergency cases.

Case Type	Case	Correctly Recognized *N* (%)	Called for Ambulance *N* (%)
Emergency Cases	Seizure	347 (66.5)	201 (38.5)
	Snake bite	451 (86.4)	108 (20.7)
	Mild chest pain	191 (36.6)	33 (6.3)
	Stroke	483 (92.5)	241 (46.2)
	Severe chest pain	453 (86.8)	197 (37.7)
	Paracetamol overdose	431 (82.6)	68 (13)
	Child head hematoma	201 (38.5)	18 (3.4)
	Potential meningococcal disease	384 (73.6)	14 (2.7)
	Older person hip pain	470 (90)	355 (68)
Non-Emergency Cases	Flu	161 (30.8)	13 (2.5)
	Older person bruising	122 (23.4)	21 (4)
	Lego in ear canal	458 (87.7)	23 (4.4)
	Stubbed toe	287 (55)	20 (3.8)
	No appetite	132 (25.3)	14 (2.7)
	Woman in labor	508 (97.3)	78 (14.9)
	Back pain	160 (30.7)	28 (5.4)
	Cut finger	98 (18.8)	11 (2.1)

## Data Availability

The data presented in this study are available on a reasonable request from the corresponding author, as it forms part of an ongoing mega project. Therefore, the data are not publicly available.

## Supplementary Materials

The following supporting information can be downloaded at https://www.mdpi.com/article/10.3390/healthcare13212801/s1, Supplementary File S1: Inter-rater agreement; Supplementary File S2: Checklist for Reporting Results of Internet E-Surveys (CHERRIES). Ref. [40] is cited in the Supplementary files.

## Abbreviations

The following abbreviations are used in this manuscript:

EMS	Emergency Medical Services
SRCA	Saudi Red Crescent Authority
IRB	Institutional Review Board
BLS	Basic Life Support

## Appendix A

**Table A1:** Predictors of correctly recognizing emergency cases and appropriately calling for an ambulance.

Variables	Recognizing Emergency Cases			Calling for Ambulance		
	Regression Coefficient (B)	95% CI for B	*p* Value	Regression Coefficient (B)	95% CI for B	*p* Value
Gender	0.004	(−0.018, 0.026)	0.730	0.003	(−0.050, 0.057)	0.902
Nationality	0.016	(−0.017, 0.050)	0.338	0.024	(−0.057, 0.105)	0.563
Age in years	0.007	(−0.016, 0.031)	0.547	−0.019	(−0.077, 0.039)	0.514
Income in SAR	−0.011	(−0.030, 0.008)	0.272	0.005	(−0.041, 0.052)	0.820
Education	0.019	(−0.002, 0.041)	0.079	0.026	(−0.027,0.078)	0.336
Studying or working in the medical field	0.010	(−0.015, 0.035)	0.424	0.038	(−0.023, 0.098)	0.220
Having a relative working in the medical field	0.002	(−0.017, 0.020)	0.864	0.044	(−0.001, 0.089)	0.057
Watching medical TV series	0.003	(−0.014, 0.020)	0.748	0.012	(−0.029, 0.053)	0.558

Statistical significance at 5%. All predictors had values of VIF below 5 with a range of 0.7 to 0.9 and tolerance values above 0.1, indicating no collinearity concerns.

## Appendix B

**Table A2:** Study participants’ demographics compared to demographics of Riyadh residents (aged 18 years and above).

Details	Study Participants N (%)	Riyadh Residents N (%)	Reference
**Gender**			[41]
Males	109 (20.9)	4,249,925 (66.73)	
Females	413 (79.1)	2,118,614 (33.27)	
**Nationality**			[41]
Saudi	478 (91.6)	2,700,570 (42.40)	
Non-Saudi	44 (8.4)	3,667,969 (57.60)	
**Age**			[41]
18–34	221 (42.3)	3,136,714 (49.25)	
35–54	214 (41)	2,605,586 (40.91)	
55+	87 (16.7)	626,239 (9.83)	
**Financial Income (SAR)**			[42]
Without a constant monthly income	85 (16.3)		
Less than 5000	118 (22.6)		
5000–10,000	103 (19.7)	SAR 4339 (average)	
10,001–15,000	86 (16.5)		
15,001–20,000	72 (13.8)		
More than 20,000	58 (11.1)		
**Education**			[43]
High School	108 (20.7)	(15.23) *	
Bachelor’s	357 (68.4)	(58.80) *	
Higher Studies	57 (10.9)	(10.22) *	

* These data are only for Saudi nationals (aged 25 years and above).

## References

[1] Morley C., Unwin M., Peterson G.M., Stankovich J., Kinsman L. (2018). Emergency department crowding: A systematic review of causes, consequences and solutions. PLoS ONE.

[2] Andrew E., Nehme Z., Cameron P., Smith K. (2020). Drivers of Increasing Emergency Ambulance Demand. Prehosp. Emerg. Care.

[3] Lowthian J.A., Cameron P.A., Stoelwinder J.U., Curtis A., Currell A., Cooke M.W., McNeil J.J. (2011). Increasing utilisation of emergency ambulances. Aust. Health Rev..

[4] Johnson K.D., Winkelman C. (2011). The effect of emergency department crowding on patient outcomes: A literature review. Adv. Emerg. Nurs. J..

[5] Powell E.S., Khare R.K., Venkatesh A.K., Van Roo B.D., Adams J.G., Reinhardt G. (2012). The relationship between inpatient discharge timing and emergency department boarding. J. Emerg. Med..

[6] McManamny T.E., Dwyer R., Cantwell K., Boyd L., Sheen J., Smith K., Lowthian J.A. (2022). Emergency ambulance demand by older adults from rural and regional Victoria, Australia. Australas. J. Ageing.

[7] Li M., Vanberkel P., Carter A.J. (2019). A review on ambulance offload delay literature. Health Care Manag. Sci..

[8] Hjälte L., Suserud B.-O., Herlitz J., Karlberg I. (2007). Initial emergency medical dispatching and prehospital needs assessment: A prospective study of the Swedish ambulance service. Eur. J. Emerg. Med..

[9] Miles J., O’Keeffe C., Jacques R., Stone T., Mason S. (2017). 17 Exploring ambulance conveyances to the emergency department: A descriptive analysis of non-urgent transports. Emerg. Med. J. EMJ.

[10] Abdul-Aziz Qawwas L., Ali Algaribi S., Al Haliq S.A., Almufareh B., Mohammed Almakhalas K. (2022). An analysis of cost and time for non-emergency calls: A retrospective study on the Emergency Medical Services resources management. J. Emerg. Med. Trauma Acute Care.

[11] Mills B., Hill M., Buck J., Walter E., Howard K., Raisinger A., Smith E. (2019). What constitutes an emergency ambulance call?. Australas. J. Paramed..

[12] Mills B., Hill M., Miles A., Smith E., Afrifa-Yamoah E., Reid D., Rogers S., Sim M. (2023). Calling an ambulance for non-emergency medical situations: Results of a cross-sectional online survey from an Australian nationally representative sample. Emerg. Med. Australas..

[13] Kirkby H.M., Roberts L.M. (2012). Inappropriate 999 calls: An online pilot survey. Emerg. Med. J..

[14] Saudi Census Population by Region, Nationality and Gender. https://www.stats.gov.sa/en/w/%D8%A7%D9%84%D8%B3%D9%83%D8%A7%D9%86-%D8%AD%D8%B3%D8%A8-%D8%A7%D9%84%D8%AC%D9%86%D8%B3%D9%8A%D8%A9?tab=436332&category=417653.

[15] Pereira S., e Silva A.O., Quintas M., Almeida J., Marujo C., Pizarro M., Angélico V., Fonseca L., Loureiro E., Barroso S. (2001). Appropriateness of emergency department visits in a Portuguese university hospital. Ann. Emerg. Med..

[16] Goldstein J., Jensen J.L., Carter A.J., Travers A.H., Rockwood K. (2015). The epidemiology of prehospital emergency responses for older adults in a provincial EMS system. Can. J. Emerg. Med..

[17] Carret M.L., Fassa A.G., Kawachi I. (2007). Demand for emergency health service: Factors associated with inappropriate use. BMC Health Serv. Res..

[18] Oktay C., Cete Y., Eray O., Pekdemir M., Gunerli A. (2003). Appropriateness of emergency department visits in a Turkish university hospital. Croat. Med. J..

[19] Ebben R.H., Castelijns M., Frenken J., Vloet L.C. (2019). Characteristics of non-conveyance ambulance runs: A retrospective study in the Netherlands. World J. Emerg. Med..

[20] Marks P., Daniel T., Afolabi O., Spiers G., Nguyen-Van-Tam J. (2002). Emergency (999) calls to the ambulance service that do not result in the patient being transported to hospital: An epidemiological study. Emerg. Med. J..

[21] Kawakami C., Ohshige K., Kubota K., Tochikubo O. (2007). Influence of socioeconomic factors on medically unnecessary ambulance calls. BMC Health Serv. Res..

[22] Fortuna R.J., Robbins B.W., Mani N., Halterman J.S. (2010). Dependence on emergency care among young adults in the United States. J. Gen. Intern. Med..

[23] Coster J.E., Turner J.K., Bradbury D., Cantrell A. (2017). Why do people choose emergency and urgent care services? A rapid review utilizing a systematic literature search and narrative synthesis. Acad. Emerg. Med..

[24] Kanstad B.K., Nilsen S.A., Fredriksen K. (2011). CPR knowledge and attitude to performing bystander CPR among secondary school students in Norway. Resuscitation.

[25] Bogle B., Mehrotra S., Chiampas G., Aldeen A.Z. (2013). Assessment of knowledge and attitudes regarding automated external defibrillators and cardiopulmonary resuscitation among American University students. Emerg. Med. J..

[26] Dong X., Kong S.Y.J., Xu H., Ho A.F.W., Blewer A.L., Birkenes T.S., Myklebust H., Zheng X., Li M., Zheng Z.-J. (2023). “Needed but lacked”: Exploring demand- and supply-side determinants of access to cardiopulmonary resuscitation training for the lay public in China. Front. Public Health.

[27] Tiwari L., Lockey A., Böttiger B.W., Rott N., Hoover A.V., Chakra Rao S., Garg R., Edara L.R. (2023). More than 302 million people reached and over 2,200,000 trained in cardiopulmonary resuscitation worldwide: The 2021 ILCOR World Restart a Heart initiative. Resusc. Plus.

[28] Kirchberger I., Heier M., Wende R., von Scheidt W., Meisinger C. (2012). The patient’s interpretation of myocardial infarction symptoms and its role in the decision process to seek treatment: The MONICA/KORA Myocardial Infarction Registry. Clin. Res. Cardiol..

[29] Shahmohamadi E., Sedaghat M., Rahmani A., Larti F., Geraiely B. (2023). “Recognition of heart attack symptoms and treatment-seeking behaviors: A multi-center survey in Tehran, Iran”. BMC Public Health.

[30] Lambert L., Brown K., Segal E., Brophy J., Rodes-Cabau J., Bogaty P. (2010). Association between timeliness of reperfusion therapy and clinical outcomes in ST-elevation myocardial infarction. JAMA.

[31] Müller U.M., Eitel I., Eckrich K., Erbs S., Linke A., Möbius-Winkler S., Mende M., Schuler G.C., Thiele H. (2011). Impact of minimising door-to-balloon times in ST-elevation myocardial infarction to less than 30 min on outcome: An analysis over an 8-year period in a tertiary care centre. Clin. Res. Cardiol..

[32] Bolanko A., Namo H., Minsamo K., Addisu N., Gebre M. (2021). Knowledge of obstetric danger signs and associated factors among pregnant women in Wolaita Sodo town, South Ethiopia: A community-based cross-sectional study. SAGE Open Med..

[33] Taghizadeh Z., Cheraghi M.A., Kazemnejad A., Pooralajal J., Aghababaei S. (2017). Difference in Perception of Pregnancy Risk in Two Maternal Age Groups. J. Clin. Diagn. Res..

[34] Uwiringiyimana E., Manirambona E., Byiringiro S., Nsanzimana A., Uhawenayo N., Ufitinema P., Bayizere J., Moreland P.J., Meharry P., Ntasumbumuyange D. (2022). Pregnant women’s knowledge of obstetrical danger signs: A cross-sectional survey in Kigali, Rwanda. PLoS Glob. Public Health.

[35] Hamam A.F., Bagis M.H., AlJohani K., Tashkandi A.H. (2015). Public awareness of the EMS system in Western Saudi Arabia: Identifying the weakest link. Int. J. Emerg. Med..

[36] Aljabri D., Albinali H. (2022). Public awareness and use of 997 emergency medical service phone number during the COVID-19 pandemic. Front. Public Health.

[37] Alanazy A., Alruwaili A., Alswaidan S., Alobaid H., Alomran A., Hzazi A., Alhussain I., Alharbi M., Binhotan M. (2024). The awareness of public about the Emergency Medical Services in the Eastern region of Saudi Arabia. PLoS ONE.

[38] Lobachova L., Brown D.F., Sinclair J., Chang Y., Thielker K.Z., Nagurney J.T. (2014). Patient and provider perceptions of why patients seek care in emergency departments. J. Emerg. Med..

[39] Koçak O., Koçak Ö.E., Younis M.Z. (2021). The Psychological Consequences of COVID-19 Fear and the Moderator Effects of Individuals’ Underlying Illness and Witnessing Infected Friends and Family. Int. J. Environ. Res. Public Health.

[40] Eysenbach G. (2004). Improving the Quality of Web Surveys: The Checklist for Reporting Results of Internet E-Surveys (CHERRIES). J. Med. Internet Res..

[41] Saudi Census Population by Detailed Age. https://www.stats.gov.sa/en/w/%D8%A7%D9%84%D8%B3%D9%83%D8%A7%D9%86-%D8%AD%D8%B3%D8%A8-%D8%A7%D9%84%D9%81%D8%A6%D8%A7%D8%AA-%D8%A7%D9%84%D8%B9%D9%85%D8%B1%D9%8A%D8%A9-%D8%A7%D9%84%D8%AA%D9%81%D8%B5%D9%8A%D9%84%D9%8A%D8%A9?tab=436332&category=417653.

[42] Saudi Census Household Income and Consumption Expenditure Statistics. https://www.stats.gov.sa/en/statistics-tabs?tab=436312&category=127329.

[43] Saudi Census Adult Skills and Learning Statistics. https://www.stats.gov.sa/en/statistics-tabs?tab=436312&category=3030916.

