# Supplementary material for: Public Recognition of Emergencies and Appropriate Ambulance Use in Riyadh: A Cross-Sectional Survey
Source: Healthcare (Basel). 2025 Nov 4;13(21):2801. doi: 10.3390/healthcare13212801 (PMC12607742; doi:10.3390/healthcare13212801)
Supplement: Supplementary file 1 [file healthcare-13-02801-s001.zip › healthcare-3898081-supplementary.pdf]

Supplementary File S1: Inter-rater agreement.

Item	Rater 1 (Yes=1 / No=0)	Rater 2 (Yes=1 / No=0)	Rater 3 (Yes=1 / No=0)	Yes_Count	No_Count	P_i	Include? (auto)
S1	0	0	1	1	2	0.333333333	1
S2	1	1	1	3	0	1	1
S3	1	1	1	3	0	1	1
S4	1	1	1	3	0	1	1
S5	1	1	1	3	0	1	1
S6	1	1	1	3	0	1	1
S7	1	1	1	3	0	1	1
S8	1	1	1	3	0	1	1
S9	1	1	1	3	0	1	1
S10	0	0	0	0	3	1	1
S11	1	1	1	3	0	1	1
S12	1	1	1	3	0	1	1
S13	1	1	1	3	0	1	1
S14	1	1	1	3	0	1	1
S15	1	1	1	3	0	1	1
S16	1	1	1	3	0	1	1

S17	1	1	1	3	0	1	1

**SUMMARY**	
**N (items with all 3 ratings)**	17
**Total ratings (N * 3)**	51
**p_yes**	0.901960784
**p_no**	0.098039216
**P¯ (mean of P_i)**	0.960784314
**P_e = p_yes^2 + p_no^2**	0.823144944
**Fleiss' Kappa (κ)**	**0.77826087**
**Interpretation**	Substantial

Supplementary File S2: Checklist for Reporting Results of Internet E-Surveys (CHERRIES).

Checklist Item	*Explanation*
Describe survey design	**Describe target population, sample frame. Is the sample a convenience sample? (In "open" surveys this is most likely.)**<br><br>*"This study included participants residing Riyadh city, Kingdom of Saudi Arabia."*<br><br>*"This study included adults (>18) who speak and read Arabic or English to maximize the chances of capturing the knowledge of both Saudi and non-Saudi nationals".*<br><br>This study involved a convenience sample.
IRB approval	**Mention whether the study has been approved by an IRB.**<br><br>*"Ethical approval from Institutional Review Board (IRB) at King Abdullah International Medical Research Centre was received (NRC23R/625/10 Oct 2023) to conduct this study."*

Informed consent	**Describe the informed consent process. Where were the participants told the length of time of the survey, which data were stored and where and for how long, who the investigator was, and the purpose of the study?**<br><br>*"All participants were informed with the study's aim and ensured that their participation is voluntarily, with no incentives offered."*<br><br>*"Informed consent was electronically attached with the survey to be signed by participants prior to starting the questionnaire."*<br><br>*"All data were stored in a computer protected password at the Emergency Medical Services Department in King Saud bin Abdulaziz University for Health Sciences, supervised by the primary investigator and accessed only by the research team. These data are not openly available but are available on request from the corresponding author. All collected data follow the retention policy by King Abdullah International Medical Research Center, allowing three years data storing."*<br><br>The informed consent included the study aim and the primary investigator name. Also, the front page of the survey contained information of the study including the time required to complete the survey, the main purpose, and the survey sections.
Data protection	**If any personal information was collected or stored, describe what mechanisms were used to protect unauthorized access.**<br><br>*"No personally identifying data were collected, and all participants were assigned with an anonymous number, to ensure the privacy of the participants."*
Development and testing	**State how the survey was developed, including whether the usability and technical functionality of the electronic questionnaire had been tested before fielding the questionnaire.**<br><br>*"This study utilized a survey developed from existing literature on similar topic and experts panel discussions.*<br>*A previously validated survey in different settings was initially used to develop the current survey [11-13]."*<br><br>*"To optimally assess the public awareness in Saudi Arabia, the survey was revised to reflect the Saudi culture and prevalent case types. A panel of expert EMS providers including three EMS assistant professors and one paramedic with over 10 years of clinical and educational experience reviewed the scenarios, resulting in the replacement of two scenarios and the modification of three responses. The inter-rater agreement was calculated using Fleiss' Kappa; the overall* $\kappa = 0.78$*, indicating substantial*

	agreement among the experts. These modifications were based on either cultural considerations (replacing 'alcohol intoxication' scenario with 'no appetite' scenario) or the low likelihood of occurrence (replacing 'box jellyfish sting' scenario with 'seizure' scenario). In addition, the responses were modified to reflect the healthcare setting in Saudi Arabia (such as replacing 'call 000 for an ambulance' with 'call 997 for an ambulance'). The revised survey was translated into Arabic, the spoken language in Saudi Arabia, by two bilingual EMS providers fluent in both languages to ensure a comprehensive understanding of the survey. The Arabic version was then backtranslated into English to ensure comprehensive understanding and equivalence of meaning."<br><br>*"Following developing the revised survey, piloting was conducted to ensure vali-dation of the new version. The final draft of the survey was distributed to another three expert EMS providers with more than 10 years of experience and a sample of the public."*<br><br>*"Based on their feedback, minor modifications were made to enhance the readability of the final draft, leading to the creation of the final version of the survey that was utilized in this study. This final version was agreed upon by the panel members through consensus and subsequently utilized in this study."*<br><br>The primary investigator tested the technical functionality of the survey prior before starting the study.
Open survey versus closed survey	**An "open survey" is a survey open for each visitor of a site, while a closed survey is only open to a sample which the investigator knows (password-protected survey).**<br><br>This study used an open survey distributed in several publicly accessible places throughout the city of Riyadh.
Contact mode	**Indicate whether or not the initial contact with the potential participants was made on the Internet. (Investigators may also send out questionnaires by mail and allow for Web-based data entry.)**<br><br>*"The digital version of the survey and printed copies were distributed in various publicly accessible places throughout the city of Riyadh. The places were previously decided to capture different demographics of Riyadh residents. The places included large shopping malls, schools, metro stations, and universities."*<br><br>No internet contact was made with the potential participants.

Advertising the survey	**How/where was the survey announced or advertised? Some examples are offline media (newspapers), or online (mailing lists – If yes, which ones?) or banner ads (Where were these banner ads posted and what did they look like?). It is important to know the wording of the announcement as it will heavily influence who chooses to participate. Ideally the survey announcement should be published as an appendix.**<br><br>*"The digital version of the survey and printed copies were distributed in various publicly accessible places throughout the city of Riyadh. The places were previously decided to capture different demographics of Riyadh residents. The places included large shopping malls, schools, metro stations, and universities."*
Web/E-mail	**State the type of e-survey (e.g., one posted on a Web site, or one sent out through e-mail). If it is an e-mail survey, were the responses entered manually into a database, or was there an automatic method for capturing responses?**<br><br>The survey barcode was distributed to the potential participants. Data was entered automatically when participants responded to the survey questions.
Context	**Describe the Web site (for mailing list/newsgroup) in which the survey was posted. What is the Web site about, who is visiting it, what are visitors normally looking for? Discuss to what degree the content of the Web site could pre-select the sample or influence the results. For example, a survey about vaccination on an anti-immunization Web site will have different results from a Web survey conducted on a government Web site.**<br><br>No Web site was used.
Mandatory/voluntary	**Was it a mandatory survey to be filled in by every visitor who wanted to enter the Web site, or was it a voluntary survey?**<br>The participation was voluntary.<br><br>*"All participants were informed with the study's aim and ensured that their participation is voluntarily, with no incentives offered."*

Incentives	**Were any incentives offered (e.g., monetary, prizes, or non-monetary incentives such as an offer to provide the survey results)?**<br><br>*"All participants were informed with the study's aim and ensured that their participation is voluntarily, with no incentives offered."*<br><br>No incentives were offered to participate in the study.
Time/Date	**In what timeframe were the data collected?**<br><br>*"This study recruited participants from November to December 2023, during which the calculated sample size was achieved."*<br><br>*"The potential participants were approached during early day time (09:00-12:00) and early evening (17:0021:00), both during weekdays and weekends."*
Randomization of items or questionnaires	**To prevent biases items can be randomized or alternated.**<br>No randomization was applied to the items, to avoid mixing between the survey's domains.
Adaptive questioning	**Use adaptive questioning (certain items, or only conditionally displayed based on responses to other items) to reduce number and complexity of the questions.**<br>N/A.
Number of Items	**What was the number of questionnaire items per page? The number of items is an important factor for the completion rate.**<br><br>The number of items were varied depending on the domain. The items were between 10 and 17 per page.
Number of screens (pages)	**Over how many pages was the questionnaire distributed? The number of items is an important factor for the completion rate.**<br><br>The whole survey contained 5 pages.

Completeness check	**It is technically possible to do consistency or completeness checks before the questionnaire is submitted. Was this done, and if "yes", how (usually JAVAScript)? An alternative is to check for completeness after the questionnaire has been submitted (and highlight mandatory items). If this has been done, it should be reported. All items should provide a non-response option such as "not applicable" or "rather not say", and selection of one response option should be enforced.**<br><br>All items in the survey were required to be answered, and respondents were unable to proceed to the next page if any item remained unanswered. If a respondent attempted to navigate away from the page without completing all items, a notification appeared to remind them of the necessity to answer all questions. Also, each question was marked with a 'star' to clearly indicate that completion was mandatory.
Review step	**State whether respondents were able to review and change their answers (e.g., through a Back button or a Review step which displays a summary of the responses and asks the respondents if they are correct).**<br><br>The survey allowed the respondents to navigate the survey through a 'back' button, which allow them to review and change their answers before final submission of the survey.
Unique site visitor	**If you provide view rates or participation rates, you need to define how you determined a unique visitor. There are different techniques available, based on IP addresses or cookies or both.**<br><br>N/A.
View rate (Ratio of unique survey visitors/unique site visitors)	**Requires counting unique visitors to the first page of the survey, divided by the number of unique site visitors (not page views!). It is not unusual to have view rates of less than 0.1 % if the survey is voluntary.**<br><br>N/A.
Participation rate (Ratio of unique visitors who agreed to participate/unique	**Count the unique number of people who filled in the first survey page (or agreed to participate, for example, by checking a checkbox), divided by visitors who visit the first page of the survey (or the informed consents page, if present). This can also be called "recruitment" rate.**<br><br>The recruitment rate was not calculated. The electronic survey was not designed to count the number of people who filled in the first survey page or the survey visitors. Also, the recruitment was conducted in different places by

first survey page visitors)	different study investigators, making it difficult to record the exact number of people who agreed to participate but did not.
Completion rate (Ratio of users who finished the survey/users who agreed to participate)	**The number of people submitting the last questionnaire page, divided by the number of people who agreed to participate (or submitted the first survey page). This is only relevant if there is a separate "informed consent" page or if the survey goes over several pages. This is a measure for attrition. Note that "completion" can involve leaving questionnaire items blank. This is not a measure for how completely questionnaires were filled in. (If you need a measure for this, use the word "completeness rate".)**<br><br>The completion rate was not calculated, as all items in the survey, including the consent form, were mandatory to be answered in order to successfully submit the responses. Therefore, we were unable to record the completion rate within the context of our study.
Cookies used	**Indicate whether cookies were used to assign a unique user identifier to each client computer. If so, mention the page on which the cookie was set and read, and how long the cookie was valid. Were duplicate entries avoided by preventing users access to the survey twice; or were duplicate database entries having the same user ID eliminated before analysis? In the latter case, which entries were kept for analysis (e.g., the first entry or the most recent)?**<br><br>No cookies were used in the survey. The user IDs were not recorded to ensure privacy and confidentiality of the respondents, and therefore we were unable to prevent multiple entries using the same user ID. However, the participants were instructed to complete the survey one time when they were approached.
IP check	**Indicate whether the IP address of the client computer was used to identify potential duplicate entries from the same user. If so, mention the period of time for which no two entries from the same IP address were allowed (e.g., 24 hours). Were duplicate entries avoided by preventing users with the same IP address access to the survey twice; or were duplicate database entries having the same IP address within a given period of time eliminated before analysis? If the latter, which entries were kept for analysis (e.g., the first entry or the most recent)?**<br><br>IP check was not used in our survey.

Log file analysis	**Indicate whether other techniques to analyze the log file for identification of multiple entries were used. If so, please describe.**<br><br>The study did not include log life analysis.
Registration	**In "closed" (non-open) surveys, users need to login first and it is easier to prevent duplicate entries from the same user. Describe how this was done. For example, was the survey never displayed a second time once the user had filled it in, or was the username stored together with the survey results and later eliminated? If the latter, which entries were kept for analysis (e.g., the first entry or the most recent)?**<br><br>There was no registration as we used open survey.
Handling of incomplete questionnaires	**Were only completed questionnaires analyzed? Were questionnaires which terminated early (where, for example, users did not go through all questionnaire pages) also analyzed?**<br><br>Only completed responses were analyzed. All items in the survey were required to be answered in order to successfully submit the responses.
Questionnaires submitted with an atypical timestamp	**Some investigators may measure the time people needed to fill in a questionnaire and exclude questionnaires that were submitted too soon. Specify the timeframe that was used as a cut-off point, and describe how this point was determined.**<br><br>Timestamp was not used.
Statistical correction	**Indicate whether any methods such as weighting of items or propensity scores have been used to adjust for the non-representative sample; if so, please describe the methods.**<br>No such methods were used in our study.<br><br>*"The data was firstly checked for normality using Shapiro–Wilk test. Descriptive analysis using mean ± standard deviation was conducted to present continuous variables, and frequencies were used for categorical variables. These findings addressed the main objective concerning assessing the public awareness. To compare responses to emergency and non-emergency scenarios across participants' demographics, independent t-test were used for gender, nationality, and variables including studying or working in medical field, having relative working in medical field, and watching medical TV series, while analysis of variance (ANOVA) test was used for age, financial income, and level of education. To identify predictors of correctly recognizing*

	emergency cases and appropriately calling for an ambulance, multiple linear regression was employed. All data were analyzed using IBM SPSS Statistics."

This checklist has been modified from Eysenbach G. Improving the quality of Web surveys: the Checklist for Reporting Results of Internet E-Surveys (CHERRIES). J Med Internet Res. 2004 Sep 29;6(3):e34 [corrected in J Med Internet Res. 2012; 14(1): e8]. Original article iw available at https://www.jmir.org/2004/3/e34/; and corrected version is available at https://www.jmir.org/2012/1/e8/. Copyright ©Gunther Eysenbach. Originally published in the Journal of Medical Internet Research, 29.9.2004 and 04.01.2012.
